# 高级别胎儿型肺腺癌并头皮转移1例

**DOI:** 10.3779/j.issn.1009-3419.2024.106.04

**Published:** 2024-02-20

**Authors:** Yangzong DEJI, Macuo BIAN, Xi WANG, Han WANG

**Affiliations:** ^1^850010 拉萨，西藏大学医学院（德吉央宗）; ^1^School of Medicine, Tibet University, Lhasa 850010, China; ^2^850034 拉萨，西藏自治区人民医院呼吸与危重症科（边玛措）; ^2^Department of Respiratory and Critical Care, Tibet Autonomous Region People's Hospital, Lhasa 850034, China; ^3^100034 北京，北京大学第一医院呼吸与危重症医学科（王玺）; ^3^Department of Respiratory and Critical Care Medicine, Peking University First Hospital, Beijing 100034, China; ^4^850034 拉萨，西藏自治区人民医院病理科（王寒）; ^4^Department of Pathology, Tibet Autonomous Region People's Hospital, Lhasa 850034, China

**Keywords:** 青年, 胎儿型肺腺癌, 头皮肿瘤, 转移, Youth, Fetal adenocarcinoma of the lung, Scalp tumor, Metastasis

## Abstract

胎儿型肺腺癌（fetal adenocarcinoma of the lung, FLAC）是一种罕见的肺部肿瘤。FLAC分为低级别FLAC（low-grade FLAC, L-FLAC）和高级别FLAC（high-grade FLAC, H-FLAC），两者在临床病理特征、生物学行为和临床结局方面有所不同。大多数H-FLAC患者是重度吸烟的中年人。本研究描述了1例罕见的非吸烟年轻男性患者，其最初表现为头顶肿块，最终被诊断为H-FLAC。本文旨在增进对FLAC的了解和认识，提高对该疾病的重视，以防止该疾病漏诊与误诊，加强早期识别、精准诊断，从而推进后续的有效治疗、改善预后。

## Abstract (en)

Fetal adenocarcinoma of the lung (FLAC) is a rare tumor. Due to its different clinicopathological features, biological behavior and clinical outcome, FLAC is classified into low-grade FLAC (L-FLAC) and high-grade FLAC (H-FLAC). Most patients with H-FLAC are middle-aged heavy smokers. Here, we describe an extremely rare case of a young male patient who denies smoking and initially presents with a mass on the top of the head and is eventually diagnosed with H-FLAC. The aim of this article is to improve the understanding and awareness of FLAC, and increase the attention to the disease, so as to prevent the underdiagnosis and misdiagnosis of the disease, strengthen early identification and accurate diagnosis, and promote subsequent effective treatment and improve prognosis.

Kradin等^[1]^首次报道了胎儿型肺腺癌（fetal adenocarcinoma of the lung, FLAC），它占肺原发肿瘤的0.1%-0.5%。由于组织病理学和临床特征的不一致，分为低级别FLAC（low-grade FLAC, L-FLAC）和高级别FLAC（high-grade FLAC, H-FLAC）两种^[2]^。L-FLAC预后较好，5年生存率可超过80%，H-FLAC预后差，多数发现时已到晚期，需要综合治疗^[2]^。2011年，国际肺癌研究协会（International Association for the Study of Lung Cancer, IALSC）、美国胸科学会（American Thoracic Society, ATS）和欧洲呼吸学会（European Respiratory Society, ERS）共同制定了一种新的肺腺癌分类，将FLAC归为浸润性腺癌的变异型^[3]^。在获得患者家属的充分知情同意和西藏自治区人民医院医学伦理委员会批准（批准编号：ME-TBHP-23-KJ-062）的情况下，本文报道西藏自治区人民医院收治的1例H-FLAC合并头皮转移病例。

## 1 临床资料

1例22岁的藏族男性患者于2022年2月出现头顶部的小结节，该结节逐渐增大，形成大约3.0 cm×2.0 cm的包块。该包块表面没有化脓或异常分泌物，且患者未出现发热或盗汗等症状。在外院接受切开引流治疗后，包块反而进一步增大，并伴有化脓和渗血现象。2022年4月，患者前来我院外科寻求治疗。胸部电子计算机断层扫描（computed tomography, CT）检查示肺部存在巨大占位病变，随后患者被转诊至呼吸内科。患者入院时的主要症状包括“头部肿物存在已2月，伴有咳血痰、呼吸困难、胸痛约10天”。在个人病史方面，患者否认吸烟和饮酒史，否认家族性肿瘤或遗传病史。患者有超过10年的生物燃料接触史，主要是牛粪。查体：体温37.7 ^o^C，脉搏116次/min，呼吸21次/min，血压123/75 mmHg，经皮血氧饱和度84%（未吸氧）。患者神志清晰，精神状态良好。头部可见到上述包块（图1）。浅表淋巴结未触及明显肿大。胸部对称，右肺中上野叩浊，呼吸音低，余肺野叩诊清晰，呼吸音清，未闻及明显的干湿性啰音。在辅助检查方面，胸部增强CT显示右上叶有包块影（图2）。头颅CT显示头顶部皮下有异常突起影，颅内未见异常。临床上考虑：（1）右肺占位性病变，恶性肿瘤的可能性较大；（2）头皮肿物性质待查。

**图1:**
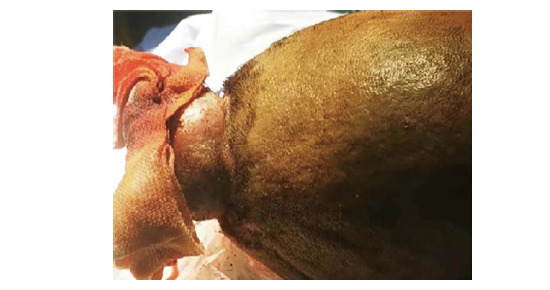
头部可见约5.0 cm×6.0 cm包块，伴有出血，无明显化脓。

**图2:**
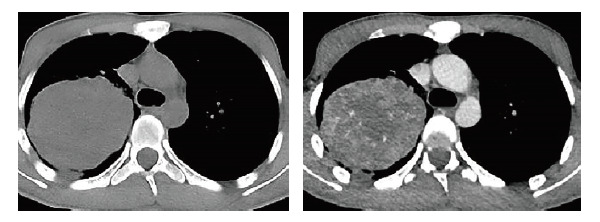
2022年4月1日胸部CT显示，右肺上叶见较大类圆形富血供囊实性包块影，右肺上叶支气管截断及并累及主支气管，病灶包绕右肺门及右主肺动脉，最大截面积约9.3 cm×7.6 cm。

患者入院后，进行了一系列的治疗和诊断操作。首先，在全麻下，通过超声支气管镜进行了针吸穿刺活检术，紧接着采用电圈套器切除了支气管内的新生物，同时进行了镜下冷冻止血（图3）。之后神经外科团队进行了头皮肿物切除术（图4）。肺组织的病理诊断结果（图5）表明，右主支气管存在恶性肿瘤，结合免疫组化结果，高度怀疑为H-FLAC，伴有坏死。具体的免疫组化结果显示上皮细胞膜抗原（epithelial membrane antigen, EMA）（局灶+），甲状腺转录因子-1（thyroid transcription factor-1, TTF-1）（部分+），神经嵴转录因子（Sox10）（-），干细胞的关键调控子（Sox-2）（-），神经胶质纤维酸性蛋白（glial fibrillary acidic protein, GFAP）（-），原癌基因ERG（ETS-related gene）（-），分子量为30-32 kDa的表面糖蛋白（p30/32MIC2）: CD99（部分+），白血病病毒整合因子1（fried leukaemia virus integration 1 gene, Fli-1）（-），B细胞白血病/淋巴瘤2基因（B-cell leukemia/lymphoma 2 gene, BCL-2）（-），Sal-like蛋白4（Sal-like protein 4, SALL4）（+）。此外，头皮肿物的病理诊断结果（图6）表明，存在转移性H-FLAC，肿瘤大小为6.0 cm×6.0 cm×4.0 cm，伴有坏死。头皮肿物的免疫组化结果包括酸性结合钙蛋白（S-100）（-），肠道黏蛋白（mucin2, MUC2）（-），磷脂酰肌醇蛋白聚糖3（glypican-3, GPC-3）（-），肠特异性转录因子（Caudal-type homeobox protein 2, CDX-2）（-），甲胎蛋白（alpha-fetoprotein, AFP）（-），人类错配修复基因家族中重要基因（mismatch repair genes MutS homolog, MSH）：MSH-2（+），MSH-6（+），错配修复蛋白MLH-1（MutL homolog-1）（+），PMS-2（post-meiotic segregation 2）（+），β-连环蛋白（β-catenin）（膜/浆+），核蛋白质（Ki-67）（70%），抑癌基因（p53）（部分弱+），突触素（synaptophysin, Syn）（-），SALL4 （+），嗜铬素A（chromogranin A, CgA）（-），神经细胞黏附分子（neural cellnadhesion molecules, CD56）（灶+），天冬氨酸转肽酶A（Napsin A）（-），TTF-1（+），细胞角蛋白7（keratin 7, CK7）（-），细胞角蛋白20（CK20）（-），波形蛋白（vimentin）（部分+），广谱细胞角蛋白抗体（AE1/AE3）（+），癌胚抗原M2A（D2-40）（部分+），分子量为145-160 kDa的III型跨膜酪氨酸激酶受体蛋白（CD117）（+），肿瘤坏死因子（CD30）（-），Sox2（±），EMA（散在+），转录因子：Oct3/4 （-），胎盘碱性磷酸酶（placental alkaline phosphatase, PLAP）（±），CD99（+）。由于患者个人原因，并未在这期间进行更全面的肿瘤基因检测。

**图3:**
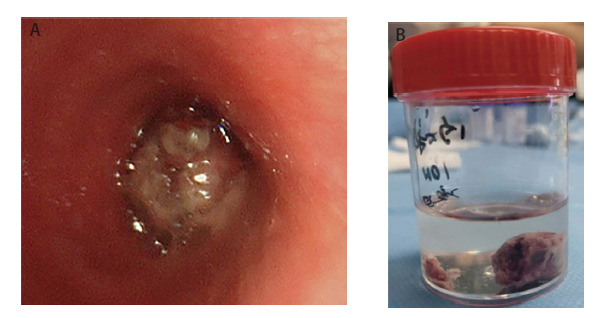
病变支气管镜下表现及切除后肿物标本。A：支气管镜下见右主支气管内新生物完全堵塞开口，白色坏死物附着，血供丰富；B：支气管镜下切除肿物直径大小约为2.0 cm×1.0 cm。

**图4:**
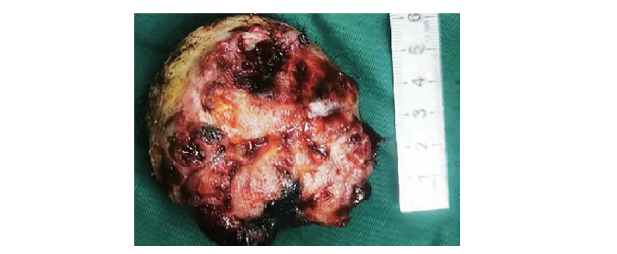
图中所见头部肿物直径大小约为6.0 cm×4.0 cm

**图5:**
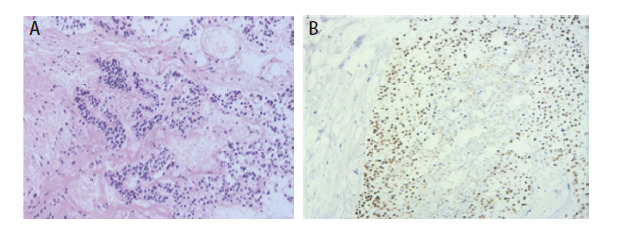
支气管内切除的肿物组织病理学检查。A：肿瘤细胞柱状，细胞核小且异型明显，胞质轻微嗜酸性，富于糖原，偶见细胞的核上、下胞质内糖原空泡，周围见坏死，缺少桑葚样结构（HE染色，×100）；B：肿瘤见大片坏死，肿瘤细胞呈复杂腺腔样排列，部分区呈筛状β-catenin：核浆异常表达（免疫组化染色，×400）。

**图6:**
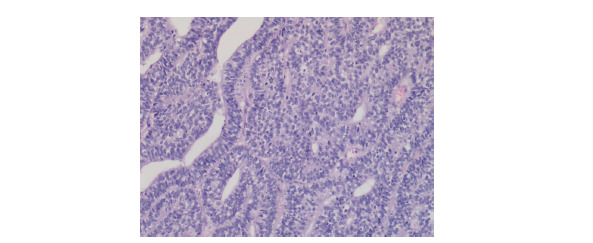
头皮肿物切除的组织病理学检查（HE染色，×100）。肿瘤细胞柱状，细胞核小且异型明显，胞质轻微嗜酸性，富于糖原，偶见细胞的核上、下胞质内糖原空泡，周围见坏死，缺少桑葚样结构。

根据病理学形态特征和免疫组化诊断，符合H-FLAC诊断标准，患者肿瘤分期为T4N3M1c，且体能状态（performance status, PS）评分为1分。患者没有化疗禁忌症。在患者及其家属的充分知情同意下，于2022年5月19日开始接受经验性EC方案（依托泊苷100 mg/m^2^+卡铂0.4 mg/m^2^）化疗，之后于2022年6月8日的化疗第2周期开始加用免疫检查点抑制剂替雷利珠单抗200 mg。在完成3个周期的化疗和2次替雷利珠单抗治疗后，患者肺部病灶未见明显增大，疗效评价为疾病稳定。然而，由于在2022年8月受到本地疫情的影响，患者未能按计划规律地接受化疗。在2022年9月17日（停化疗后72 d），患者因为“右侧胸部疼痛伴气短1月”入院。胸部CT显示右肺病灶进展（图7A），在患者及其监护人的充分知情同意下，决定给予单纯多西他赛（75 mg/m^2^）化疗。2022年11月1日，患者因“咯血1天”再次就诊，胸部CT显示右肺肿块明显增大（图7B）；同时，患者的炎症指标明显升高，采取抗感染、镇痛等对症治疗措施。2022年11月6日，患者突发意识丧失，经抢救无效最终不幸离世。

**图7:**
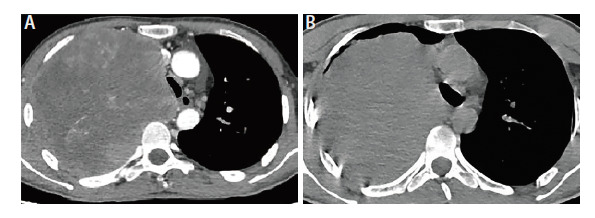
胸部CT。A：肿块最大截面约为13.0 cm×12.0 cm（2022年9月17日）；B：肿块最大截面积约为14.4 cm×15.9 cm（2022年11月4日）。

## 2 讨论

H-FLAC主要发生在中老年男性重度吸烟者中，男女患病比例为33:3，即男性患者占92%，其中93%的患者有吸烟史^[2]^。由于早期H-FLAC几乎没有特定的症状和体征，通常难以及时发现。许多患者是在常规体检时意外被诊断出H-FLAC，而此时往往已经发生淋巴结或远处转移^[4]^。肺癌通常通过淋巴结和血液传播扩散，其中肝脏、颅内和骨骼是最常见的转移部位。然而，H-FLAC的扩散可能涉及到胸膜、肝脏、卵巢、眼睛和皮肤等部位^[2,5]^。

在临床病理特征上，H-FLAC一般不表现出桑葚体，但其细胞核异型性更加明显，核仁肥大，且频繁出现有丝分裂。H-FLAC还可能转变为常见腺癌形态，出现坏死等特异性表现^[3]^。肺部至少有50%的胎儿形态，同时混合高级别神经内分泌癌、肠腺癌和绒毛膜癌^[2]^。免疫组织化学染色，可以分析H-FLAC的表达特点。在H-FLAC中，β-catenin多表现在胞膜而非胞核，约50%的病例伴有神经内分泌分化，Syn、CgA和CD56的阳性率可达30%-60%。相关肿瘤标志物AFP和GPC-3在H-FLAC中的阳性率约为90%，尤其是在混合型中^[2,6]^。基因特征方面，与常规肺腺癌相比，H-FLAC中p53基因的过度表达更为明显，而表皮生长因子受体（epidermal growth factor receptor, EGFR）、鼠类肉瘤病毒癌基因（kirsten rat sarcoma viral oncogene, KRAS）的突变频率则相对较低^[7]^。Suzuki等^[8]^对16例H-FLAC病例的全外显子基因测序中发现，7例p53高突变，6例KMT2C又称混合谱系白血病基因3（mixed lineage leukemia 3, MLL3）高突变，4例KRAS高突变，3例神经蛋白基因：NF1高突变，3例丝氨酸/苏氨酸激酶11（STK11）高突变，2例编码β-catenin基因CTNNB1高突变，1例EGFR高突变，5例病例表现低程序性死亡受体1（programmed cell death protein 1, PD-1）。

在诊断和治疗方面，目前主要依靠病理学和免疫组织化学特征来确诊H-FLAC。对于大多数H-FLAC患者，在被发现时通常已处于晚期且恶性程度较高，因此需要采用手术、化疗和放疗的综合治疗方法。尽管远期疗效较好，但纯H-FLAC的预后仍然不容乐观^[9,10]^。在本病例中，患者最初因头皮肿物就诊，通过常规胸部CT检查才发现肺部病变。头部肿物经过病理诊断后被认为是转移瘤。肺部活检显示癌细胞排列成复杂的腺腔样结构，细胞核异型性明显，核下空泡存在，但没有典型的桑葚体，β-catenin的免疫染色显示胞膜呈阳性，同时p53也表达，这符合H-FLAC的特征。由于患者的经济和地理医疗条件的限制，未能进行更全面的基因检测和肿瘤免疫检测。因此，他接受了经验性治疗，包括3次EP方案化疗和2次免疫检查点抑制剂治疗，疗效评估为疾病稳定。然而，由于疫情的影响，患者未能按计划进行化疗，导致疾病进展，肺部病变不断扩大，最终压迫气道导致患者不幸过世。

尽管这一病例的肺部原发病灶较大，但最初的临床表现是头部肿物，这在临床上可能导致病情延误和误诊。通过报道本病例并复习相关文献，我们希望提高临床医生对这种罕见疾病的认识，减少诊断延误，强调早期发现和综合治疗的重要性，以提高患者的生存率和改善预后。此外，深入研究H-FLAC的免疫组织化学和基因特征将有助于更好地理解这种肿瘤的发展和治疗机制，为临床实践提供更有力的指导。

**Competing interests**

The authors declare that they have no competing interests.
